# Transthoracic echocardiography of left ventricular underfilling improves risk stratification in pulmonary arterial hypertension

**DOI:** 10.1038/s41598-025-28206-z

**Published:** 2025-12-04

**Authors:** Ashfaq Ahmad, Songlin Zhang, Qian Ren, Lingling Li, Yajuan Du, Xiaoyu Wang, Ting Liu, Ekhlas Mahmoud Al-Hashedi, Fenling Fan

**Affiliations:** 1 Department of Cardiovascular Medicine, First Affiliated Hospital, Xi’an Jiaotong University, Xi’an, 710061 Shaanxi China; 2 Sunsimiao Hospital, Beijing University of Chinese Medicine, Beijing, China

**Keywords:** Transthoracic echocardiography, Current LV model, Left ventricular underfilling, Pulmonary arterial hypertension, Risk stratification, Cardiology, Medical research

## Abstract

Pulmonary arterial hypertension (PAH) is characterized by right ventricular (RV) adaptation to elevated afterload. However, the prognostic value of left ventricular (LV) underfilling in response to RV volume overload is unknown. We aimed to investigate the prognostic value of LV underfilling in PAH. Does Trans-thoracic echocardiography (TTE) assessed LV underfilling enhance prognostic accuracy in PAH beyond existing models such as REVEAL-Lite 2.0 and REVEAL-ECHO? 270 patients with PAH were prospectively enrolled. LV underfilling was defined as the LV volume-to-mass (V/M) ratio of < 0.8 ml/g. TTE and hemodynamic parameters were compared between patients with and without LV underfilling. The Cox regression model identified LV parameters significantly predictive of survival. The current LV model was developed incorporating LV parameters and was compared with established models, including REVEAL-Lite 2.0 and REVEAL-ECHO. Cox-regression and model performance metrics, including the C-index, Akaike Information Criterion (AIC), Net Reclassification Improvement (NRI), and Integrated Discrimination Improvement (IDI), were used to assess the model’s prognostic accuracy. Patients with LV underfilling exhibited more advanced disease, with higher hemodynamic indices and impaired RV function (all p < .0001). Over a median 29.8 months follow-up, LV underfilling was associated with worse survival (Log-rank p < .0001). LV underfilling (HR: 5.32, 95% CI; 3.05–9.28, p = < .0001), RV/LV-basal diameter (HR: 1.96, 95% CI; 1.066–3.624, p = 0.03), LV end-diastolic diameter (HR: 1.071, 95% CI: 1.030–1.114, p = 0.0005) served as independent predictors of adverse outcomes. The current LV model integrating LV parameters demonstrated superior discrimination (Mean C-index: 0.716 ± 0.06) vs REVEAL-Lite 2.0 (Mean C-index: 0.585 ± 0.07) and REVEAL-ECHO (Mean C-index: 0.717 ± 0.11). The model also demonstrated superior reclassification and discrimination performance compared to traditional models (NRI: 0.225, IDI: 2.28). TTE-assessed LV underfilling is a novel, valuable prognostic marker in PAH. The Current LV model, in addition to REVEAL-ECHO, offers enhanced prognostic capabilities for PAH management and may serve as a key tool in optimizing PAH patient care.

**Supplementary Information:**

The online version contains supplementary material available at 10.1038/s41598-025-28206-z.

## Introduction

Pulmonary arterial hypertension (PAH) is a progressive, life-threatening condition characterized by elevated pulmonary vascular resistance (PVR) and right ventricular (RV) failure^1,2^, leading to significant morbidity and mortality^2–8^. A study by D’Alonzo et al.^9^ PAH revealed that survival was closely associated with cardiac index (CI) and right atrial (RA) pressure. Since then, several imaging biomarkers for RV failure, such as tricuspid annular plane systolic excursion (TAPSE)^7–11^, RV end systolic measurements, RV free wall longitudinal strain (RVFWS), and RA size and function have proven event-free survival in PAH^3,12–15^.

Left ventricular (LV) underfilling, characterized by reduced LV volume without a proportional decrease in mass, may serve as a critical marker of disease severity and adverse outcomes in PAH. Although RV hypertrophy and dilation are well-documented adaptive responses to increased afterload in PAH^16–18^ but the role of LV remodeling, particularly LV underfilling in response to this afterload, remains unknown. Recent studies have demonstrated that not all evidence of RV concentric hypertrophy is adaptive in PAH patients^19^. The adaptive nature of this should be interpreted with ventricular interdependence^20^. The connection between ventricles is crucial, as the circulatory system is continuous, and both ventricles are interdependent via the shared septum and common pericardial space^1,21^. Functional ventricular interdependence, defined as the mechanical coupling of the two ventricles via the interventricular septum, myocardial fibers, and pericardial constraint, modulates LV diastolic filling in PAH. RV pressure overload causes septal shift and pericardial restraint, directly compressing the LV and reducing preload, as evidenced from magnetic resonance imaging and echocardiographic studies^22,23^. Reduced LV preload, in turn, predicts adverse outcomes in PAH^19^. Given this mechanistic backdrop, the LV end-diastolic volume-to-mass ratio provides a physiologically grounded and potentially prognostic marker of LV underfilling in PAH. Recent imaging work also demonstrates that RV dysfunction parallels LV strain impairment, further underscoring the interplay captured by this index^24^. Moreover, studies suggest that prolonged pressure overload causes inadequate filling of the LV^19,22^, potentially leading to atrophic remolding of the LV^19^.

Although LV underfilling has been noted in PAH^19^, the prognostic significance of this phenomenon, especially regarding ventricular interdependence, remains unexplored. The recently developed REVEAL-ECHO risk model, using echocardiographic parameters, effectively stratifies PAH patients with strong discrimination^25^. However, the REVEAL-ECHO score assesses severity and high-risk groups using only RV enlargement, systolic dysfunction, tricuspid regurgitation, and pericardial effusion, and hence is limited to left heart interaction and underfilling. We aimed to (i) evaluate echocardiography’s role in detecting LV underfilling and its prognostic significance in PAH, (ii) develop a robust prognostic model integrating LV parameters with REVEAL-ECHO indices for enhanced risk stratification, and (iii) compare the incremental predictive power of this model against established risk tools, including Registry to Evaluate Early and Long-Term PAH Disease Management (REVEAL 2.0 calculator) abridged version, REVEAL-Lite 2.0.

## Study design and methods

### Patient selection

**Fig. 1:**
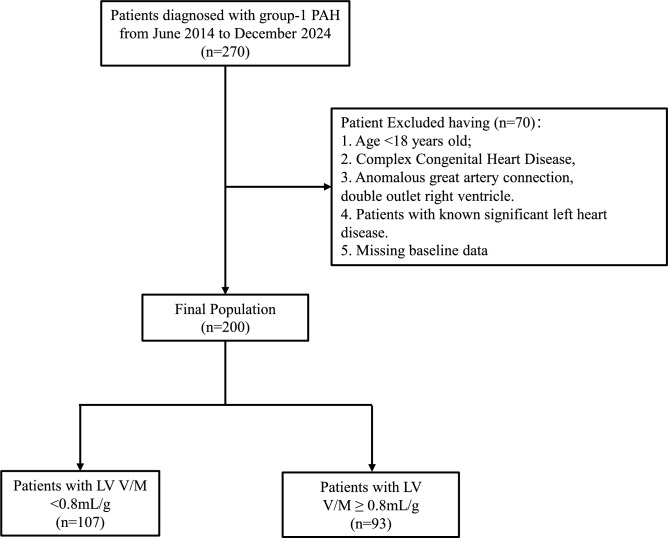
Study the flow chart.

A total of 270 group-1 PAH patients from June 2014 to December 2024 were prospectively enrolled from the Department of Cardiovascular Medicine, the First Affiliated Hospital of Xi’an Jiaotong University (Fig. 1). PAH diagnostic criteria were according to the 2022 European Society of Cardiology/European Respiratory Society (ESC/ERS) guidelines^6^. The inclusion criteria were (i) patients with a mean pulmonary artery pressure (mPAP) ≥ 20 mmHg, pulmonary artery wedge pressure (PAWP) ≤ 15 mmHg, and PVR of > 3 Wood-units; (ii) patients who underwent transthoracic echocardiography (TTE) within 48 h of right heart catheterization (RHC); and (iii) patients with aged ≥ 18 years. Exclusion criteria involved: (i) patients with complex congenital heart defects (single ventricle, single atrium, complete atrioventricular septal defects, transposition of the great arteries, double-outlet right ventricle); (ii) individuals under 18 years old; (iii) patients with known significant left heart disease including moderate or severe left-sided valvular disease, left ventricular systolic dysfunction (LVEF < 50%), hypertrophic or restrictive cardiomyopathies; and (iv) those with missing data. The study was approved by the institutional ethics committee of the First Affiliated Hospital of Xi’an Jiaotong University, all methods were performed in accordance with the relevant guidelines and regulations, and all patients were provided with informed consent.

### Right heart catheterization

RHC was performed by experienced faculty (F.F. & S.Z.) in our institution’s cardiac catheterization lab, following standard guidelines, to obtain hemodynamic measurements for diagnosis and severity assessment of PAH^26^. Under local anesthesia, introducer sheaths were inserted into the femoral vein and artery. A catheter advanced via the femoral vein accessed the inferior vena cava (IVC), superior vena cava, right atrium and ventricle, major pulmonary arteries, left atrium (LA) and ventricle, pulmonary veins, and aorta. This setup minimized time-consuming catheter manipulations and enabled simultaneous monitoring of pressures, including right ventricular systolic pressure (RVSP). Intravascular pressure was tracked using fluid-filled transducers. Pulmonary and systemic blood flow, cardiac output (CO), and CI were calculated using the Fick principle with assumed oxygen consumption^27,28^. Common formulas [PVR = (mPAP-PAWP)/CO] were used to calculate PVR^29^.

### Transthoracic echocardiography

TTE was performed by an experienced cardiologist (D.Y.) using a GE Vivid E9 system with a 5.1-MHz transducer (GE, Fairfield, CT, USA), following international guidelines^30,31^. Echocardiographic dimensional and volumetric measurements were performed according to ASE/EACI standards, using two-dimensional and M-mode techniques. Reduced LV chamber size was defined as an end-diastolic diameter (LVEDD) below 42 mm in males and 37.8 mm in females. Similarly, an enlarged LV chamber size was defined as an LVEDD exceeding 58.4 mm and 52.2 mm in males and females, respectively^32^. LV mass was calculated using the standard formula [LV mass (g) = 0.8 × [1.05(IVST + LVID + PWT)^3^ − (LVID)^3^] + 0.6^33^. LV volumes were measured using Biplane disc summation methods^32^. The LV end-diastolic volume-to-mass ratio (LV V/M, ml/g) was employed to classify patients into LV underfilling (< 0.8 ml/g) and non-underfilling (≥ 0.8 ml/g) groups, using previously established sex-specific normal values^34^.

### Longitudinal follow-up and clinical outcomes

Patient follow-up was conducted through scheduled clinical evaluations or telephone interviews every 3–6 months post-baseline, following clinical guidelines^6^. The primary endpoints included all-cause mortality, heart failure (HF) rehospitalizations, and escalation of HF-related medications, indicative of disease progression. Follow-up spanned from the date of RHC to the occurrence of an event or the final patient contact.

### Model development and validation

In the initial phase, predictors were selected based on clinical relevance and study objectives. Subsequently, a penalized regression approach was adopted using the Least Absolute Shrinkage and Selection Operator (LASSO) to select a parsimonious set of predictors from the candidate variables. To minimize prediction error, LASSO regression was implemented using tenfold cross-validation to determine the optimal penalty parameter (λ). This method reduces overfitting by shrinking the coefficients of less important variables to zero, thereby enhancing model generalizability. To assess agreement between observed outcomes and predicted risks, internal model calibration was conducted using a bootstrapped calibration curve (1,000 repetitions), comparing predicted vs. observed survival probabilities at 12, 24, and 36 months. Integrated Discrimination Improvement (IDI) and Net Reclassification Improvement (NRI) were used to quantify the added prognostic value of the model compared with the established models REVEAL-Lite 2.0 and REVEAL-ECHO, computed using the same dataset, based on its predefined parameters. The IDI and NRI were computed at a fixed 1-year time point (t₀ = 1), with statistical inference based on 1,000 perturbation resamples to ensure robustness. Mean Harrell’s concordance index (C-index) was used for model discrimination and parsimony, and corrected Akaike Information Criterion (AIC) and Bayesian Information Criterion (BIC) for model selection. Net-benefit curves for all models were assessed across threshold probabilities from 0.00 to 1.00 to evaluate clinical utility. Time-dependent receiver operating characteristic (ROC) curves were generated to compare the predictive accuracy of models.

### Reproducibility

Intra-and inter-observer reproducibility were assessed for LV parameters used in the current LV model. For intra-observer variability, 30 randomly selected patients’ measurements were obtained blindly by the same observer over a period of 3–4 days. For inter-observer variability, the same 30 patients’ measurements were carried out by another independent observer in a completely blinded manner. Coefficients of variation (CoV) evaluate variability, quantified as the absolute differences between the two repeated measurements in percentages of their mean, and the intraclass correlation coefficient (ICC).

### Statistical analysis

All statistical analyses were performed using R (version 4.4.3). The normality test was conducted using the Shapiro–Wilk test, with continuous data expressed as mean ± standard deviation (SD) or median (interquartile range [IQR]). Categorical variables were expressed as proportions (%). For comparison, the chi-square test was used for categorical variables, whereas the independent sample t-test or Mann–Whitney U-test was used for continuous variables. Bivariate correlations were tested with Pearson’s correlation coefficient for normally distributed data and Spearman’s rank correlation coefficient for non-normally distributed data. To account for skewed distributions, N-terminal pro-brain natriuretic peptide (NT-proBNP) levels were log-transformed before analysis.

Time-to-event of interest analysis was performed using the Kaplan–Meier method, and differences in survival between groups were evaluated using the log-rank test. The Cox proportional hazard regression model was used to estimate hazard ratios (HRs) with 95% confidence intervals (CIs) in both univariate and multivariate contexts. Time-dependent ROC curves were generated using the “pROC” package. IDI and NRI to quantify the added prognostic value of the model compared with the established models were tested using the “survIDINRI” package.

## Results

### Patient demographic and clinical characteristics

Table 1 shows the demographic and clinical characteristics of the study population. The study results comprised 200 subjects divided into two groups: 107 with LV underfilling and 93 without LV underfilling, with a mean age of 38 ± 14, with no significant difference between the two groups (p = 0.1). Gender distribution, height, weight, and other baseline clinical parameters were comparable between the groups (p > 0.05 for all). NT-proBNP levels were significantly elevated in the LV underfilling group (1326 pg/ml vs 383 pg/ml, p = 0.01), with a higher proportion of patients in WHO functional class-IV (42.9% vs 26.8%, p < 0.001). RV-PA coupling, assessed by the surrogate marker TAPSE to systolic pulmonary artery pressure (sPAP) ratio, was significantly reduced in the LV underfilling group (p < 0.0001).

**Table 1:** Demographic and clinical characteristics of all subjects.

**Variables**	**All (n = 200)**	**LV-underfilling (n = 93)**	**Without LV-underfilling (n = 107)**	***P* value**
Age (Years)	38 ± 14	39 ± 14	41 ± 14	0.1
Gender (male/female)	75/125	44/63	31/62	0.63
Height (m)	1.60 ± 0.08	1.64 ± 0.07	1.62 ± 0.09	0.971
Weight (kg)	57 ± 15	56 ± 15	57 ± 15	0.94
Body mass index (kg/m2)	21.4 ± 5.3	21.3 ± 5.5	21.4 ± 5.1	0.979
Body surface area (m^2^)	1.6 ± 0.23	1.5 ± 0.23	1.5 ± 0.24	0.962
Heart rate (bpm)	86 ± 13	87 ± 13	84 ± 14	0.355
Systolic blood pressure (mmHg)	116 ± 16	116 ± 16	121 ± 17	0.472
Diastolic blood pressure (mmHg)	76 ± 14	78 ± 14	76 ± 12	0.71
6MWD (m)	345 ± 87.4	326 ± 80.4	469 ± 93.3	0.952
NTproBNP (pg/mL)	790 (230 to 2106)	1326 (299 to 2546)	383 (133 to 1447)	0.01
REVEAL-Lite 2.0 Score	3.04 ± 1.88	3.81 ± 1.75	2.15 ± 1.62	< 0.0001
WHO functional class, n (%)				< 0.001
I	12 (6.0)	3 (2.8)	9 (9.6)	
II	82 (41.0)	32 (29.9)	50 (53.7)	
III	71 (35.5)	46 (42.9)	25 (26.8)	
IV	35 (17.5)	26 (24.2)	9 (9.6)	
**PAH Severity, n (%)**				< 0.0001
Mild	43 (21.5)	3 (2.8)	40 (43.0)	
Moderate	56 (28.0)	22 (20.5)	34 (36.5)	
Severe	101 (50.5)	82 (76.6)	19 (20.4)	
**PAH etiology, n (%)**				< 0.001
CHD-PAH	121(60.5)	63(58.8)	58(62.3)	
IPAH	35(17.5)	22(20.5)	13 (13.9)	
CTD-PAH	44 (22.0)	22(20.5)	22 (23.6)	
**Right heart catheterization**				
RAP (mmHg)	8.0 (5.0 to 12.3)	9.4 (5.0 to 13.0)	7.0 (5.0 to 11.0)	0.02
RVSP (mmHg)	31.0 (24.0 to 36.5)	33.0 (31.0 to 39.0)	25.0 (19.0 to 31.0)	< 0.0001
mPAP (mmHg)	49.5 (32.0 to 66.0)	62.0 (50.0 to 75.0)	33.0(28.0 to 38.5)	< 0.0001
PVR (Wood-unit)	8.5 (3.8 to 13.7)	11.2 (6.4 to 17.9)	6.3 (3.3 to 6.60)	< 0.0001
SVR (Wood-unit)	21.8 (13.9 to 27.1)	22.4 (15.7 to 27.4)	21.3 (10.5 to 25.9)	0.374
PCWP (Wood-unit)	11.0 (6.4 to 13.6)	12.0 (7.7 to 15.0)	10.0 (6.0 to 13.0)	0.129
CO (L/min)	3.6 (3.20 to 5.98)	3.6 (3.1 to 5.5)	3.7 (3.2 to 6.2)	0.323
CI (L/min/m^2^)	2.3 (1.93 to 3.51)	2.3 (1.8 to 3.4)	2.5 (1.9 to 3.8)	0.46
SvO2 (%)	68.5 (62.0 to 72.2)	62.5 (56.5 to 71.5)	71.5 (64.0 to 72.8)	0.353
Qp/Qs	1.2 (0.83 to 2.04)	1.1 (0.8 to 2.1)	1.3 (0.86 to 2.04)	0.312

6MWD; 6-min walking distance; CHD-PAH: congenital heart disease-related pulmonary arterial hypertension; CTD-PAH; connective tissue disease-related pulmonary arterial hypertension; CI: cardiac index; CO: cardiac output; IPAH: idiopathic pulmonary arterial hypertension; mPAP: mean pulmonary artery pressure; PCWP: pulmonary capillary wedge pressure; PVR: pulmonary vascular resistance; RAP: right atrial pressure; RVSP: right ventricular systolic pressure; SVR: systemic vascular resistance; SvO2: venous oxygen saturation; WHO: world health organization.

Significant structural and functional differences were observed between patients with and without LV underfilling, detected by TTE. Patients with LV underfilling had smaller LV end-diastolic volume (48.7 vs. 61.4 ml, p < 0.0001), end-systolic volume (19.2 vs. 21.2 ml, p = 0.008), LVEDD (40.0 vs. 50.0 mm, p < 0.0001), and LVESD (29.0 vs. 35.0 mm, p < 0.001). RV functional parameters indicated significant deterioration in the LV underfilling group, as evidenced by a higher sPAP (98.0 mmHg vs 76.0 mmHg, p < 0.0001) and reduced TAPSE (18.0 mm vs 21.0 mm, p < 0.0001). RV-PA and RV-LV coupling were also significantly reduced, indicating impaired biventricular interaction (p < 0.0001). Additionally, LA end-systolic diameter was smaller (30.0 vs. 33.0 mm, p = 0.009), while RA end-systolic and IVC diameters were larger, reflecting elevated right heart pressure overload (p = 0.002 and 0.013, respectively) (Table 2).

**Table 2:** Trans-thoracic echocardiographic parameters among the cohort.

Variables	All (n = 200)	LV-underfilling (n = 107)	Without LV-underfilling (n = 93)	*P* value
AOA (mm)	27.0 (25.0 to 30.0)	27.0 (25.0 to 30.0)	29.0 (25.5 to 32.0)	0.247
MPA (mm)	28.0 (25.0 to 33.0)	27.0 (25.0 to 33.0)	28.0 (25.0 to 32.0)	0.917
LAESTD (mm)	31.5 (29.0 to 33.0)	30.0 (28.0 to 33.0)	33.0 (30.0 to 36.0)	0.0009
LVEDD^(Lt-Rt)^ (mm)	46.0 (39.0 to 51.0)	40.0 (38.0 to 46)	50.0 (47.0 to 53.0)	< 0.0001
LVEDD^(A-P)^ (mm)	45.0 (38.0 to 51.0)	38.8 (35.9 to 43.8)	49.0 (45.7 to 52.8)	<.0001
LVESD^(Lt-Rt)^ (mm)	33.0 (31.0 to 36.0)	29.0 (26.0 to 31.0)	35.0 (33.0 to 39.4)	<.0001
LVESD^(A-P)^ (mm)	32.0 (29.3 to 35.0)	28.0 (25.4 to 30.0)	34.2 (32.0 to 38.0)	<.0001
LVEDV (ml)	52.1 (31.9 to 66.8)	48.7 (39.5 to 57.9)	64.1 (59.1 to 79.4)	< 0.0001
LVEDVi (ml/m^2^)	32.8 (26.6 to 41.1)	30.1 (25.4 to 35.4)	41.0 (34.5 to 46.9)	< 0.0001
LVESV (ml)	20.0 (15.0 to 27.0)	19.2 (13.6 to 26.4)	21.2 (17.7 to 28.8)	0.008
LVESVi (ml/m^2^)	12.2 (9.3 to 16.1)	11.6 (8.7 to 15.8)	13.3 (10.8to16.6)	0.01
LV-mass (g)	71.2 (62.7 to 87.7)	78.2 (65.2 to 100.2)	72.8 (64.0 to 88.1)	0.04
LV-mass index (g/m^2^)	44.4 (38.6 to 54.3)	47.2 (41.6 to 58.2)	45.3 (39.0 to 53.4)	0.03
LVEF (%)	66.0 (64.3 to 68.0)	66.0 (64.0 to 68.0)	67.0 (64.5 to 68.5)	0.759
LV V/M (ml/g)	0.75 (0.59 to 0.88)	0.53 (0.33 to 0.65)	0.89 (0.83 to 1.03)	< 0.0001
FAC (%)	34.5 (31.0 to 37.0)	32.0 (31.0 to 35.0)	37.0 (35.0 to 39.0)	< 0.0001
EI	1.03 (0.98 to 1.07)	1.05 (1.03 to 1.09)	0.98 (0.98 to 1.04)	< 0.0001
RAESTD (mm)	36.0 (33.0 to 50.0)	37.0 (33.0 to 51.0)	36.0 (33.0 to 50.0)	0.823
RVEDTD, basal (mm)	33.0 (31.0 to 38.0)	36.0 (32.0 to 39.0)	33.0 (30.0 to 36.5)	0.006
sPAP (mmHg)	90.0 (70.0 to 108.0)	98.0 (81.0 to 117.0)	76.0 (55.0 to 102.0)	< 0.0001
TAPSE (mm)	19.0 (17.0 to 22.0)	18.0 (16.0 to 19.0)	21.0 (20.0 to 23.0)	< 0.0001
RV/LV-basal ratio	0.73 (0.65 to 1.02)	0.86 (0.71 to 1.10)	0.66 (0.59 to 0.72)	< 0.0001
RV-PA coupling	0.27 (0.16 to 0.29)	0.18 (1.14 to 0.22)	0.28 (0.21 to 0.39)	< 0.0001
IVC (mm)	20.0 (17.0 to 22.0)	22.0 (20.8 to 23.0)	16.0 (13.0 to 18.0)	< 0.0001
PE (%)	84 (42.0)	65 (60.7)	19 (20.4)	< 0.0001
PA/Ao	1.0 (0.88 to 1.15)	1.0 (0.88 to 0.80)	0.96 (0.90 to 1.14)	0.442

AOA: ascending aorta; EI: eccentricity index; FAC: fractional area change; IVC: inferior vena cava; LAESTD: left atrium end systolic transverse diameter; LV: left ventricle; LVEDD^(A-P)^: left ventricular end-diastolic diameter (anteroposterior); LVEDD^(Lt-Rt)^: left ventricular end-diastolic diameter (left–right); LVESD^(A-P)^: left ventricular end-systolic diameter (anteroposterior); LVESD^(Lt-Rt)^: left ventricular end-diastolic diameter (left–right); LVEDV: left ventricular end-diastolic volume; LVEDVi: left ventricular end-diastolic volume index; LVESV: left ventricular end-systolic volume; LVESVi: left ventricular end-systolic volume index; LVEF: left ventricular ejection fraction; LV V/M: left ventricular volume-to-mass ratio; MPA: main pulmonary artery; PA/Ao pulmonary artery-to-aorta ratio; PE: pericardial effusion; RAESTD: right atrium end-systolic transverse diameter; RV: right ventricle; RVEDTD: right ventricular end-diastolic transverse diameter; sPAP: systolic pulmonary artery pressure; TAPSE; tricuspid annular plane systolic excursion.

### LV underfilling vs functional and hemodynamic parameters

**Fig. 2:**
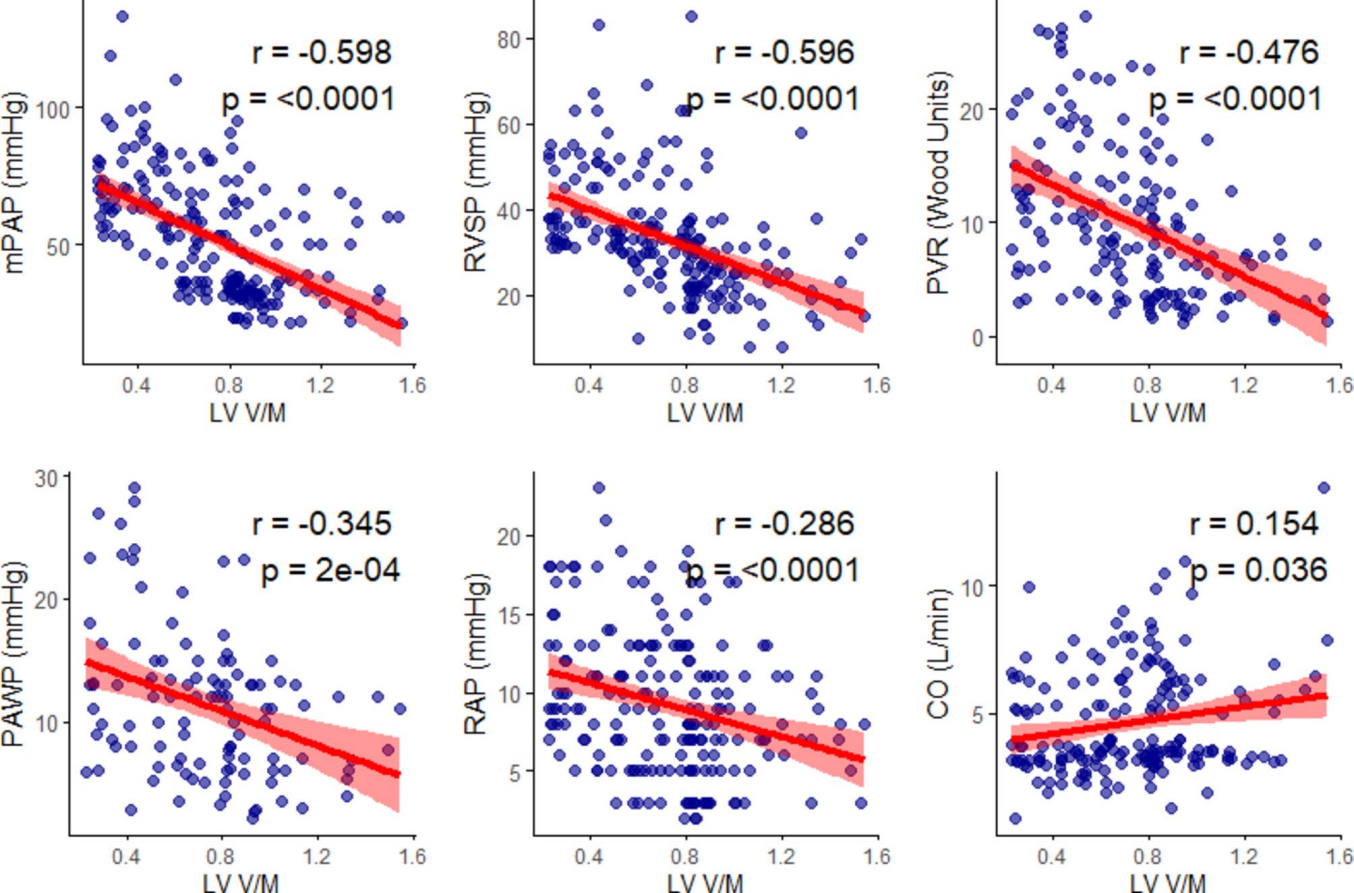
Spearman’s correlation analysis of hemodynamic parameters with LV volume-to-mass ratio, evaluating associations between hemodynamics and LV-remodeling. CO: cardiac output; mPAP: mean pulmonary artery pressure; PVR: pulmonary vascular resistance; PCWP: pulmonary capillary wedge pressure; RAP: right atrial pressure; RVSP: right ventricular systolic pressure.

Figure 2 depicts the relationship between the LV V/M and the RHC hemodynamic parameters. A significant negative correlation was observed between LV V/M and mPAP (r = − 0.598), RVSP (r = − 0.596), and PVR (r = − 0.476), all p < 0.0001. A weak but significant negative correlation was found between LV V/M and PAWP (r = − 0.345, p = 2e-04) and RAP (r = − 0.286, p = 0.036). No significant correlation was observed between LV V/M and CO.

**Fig. 3:**
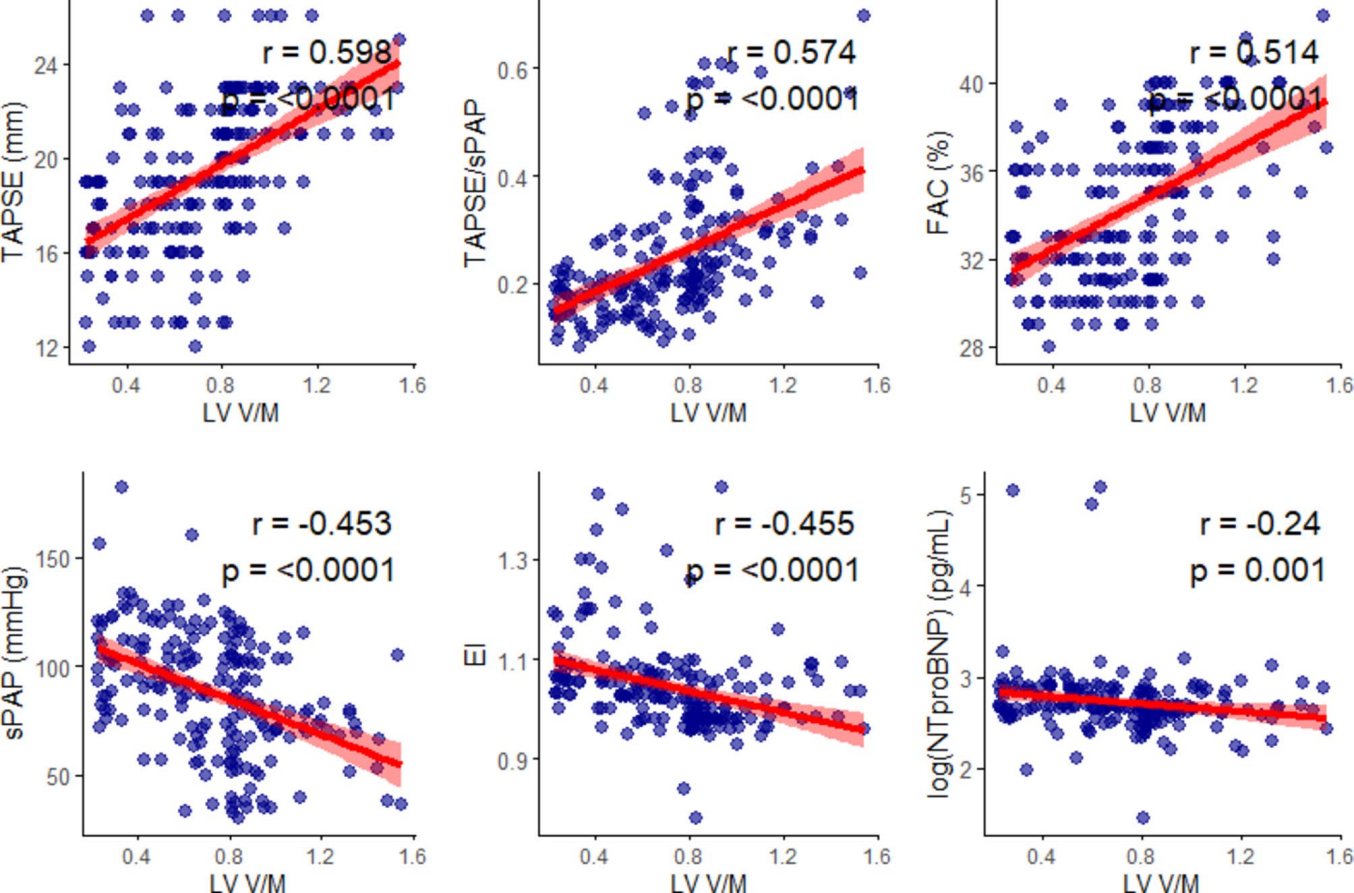
Spearman’s correlation analysis of RV-functional parameters with LV volume-to-mass ratio, assessing relationships between RV performance and LV-remodeling. FAC: fractional area change; sPAP: systolic pulmonary artery pressure; TAPSE: tricuspid annular plane systolic excursion.

Similarly, RV-functional parameters such as TAPSE, fractional area change (FAC), and RV-PA coupling were significantly positively correlated with LV V/M (r = 0.598, 0.514, and 0.574, all p < 0.0001, respectively), whereas sPAP, eccentricity-index (EI), and logNT-proBNP were negatively correlated with LV V/M. (Fig. 3).

### Comparison of hemodynamic and RV-functional parameters stratified by LV underfilling

**Fig. 4:**
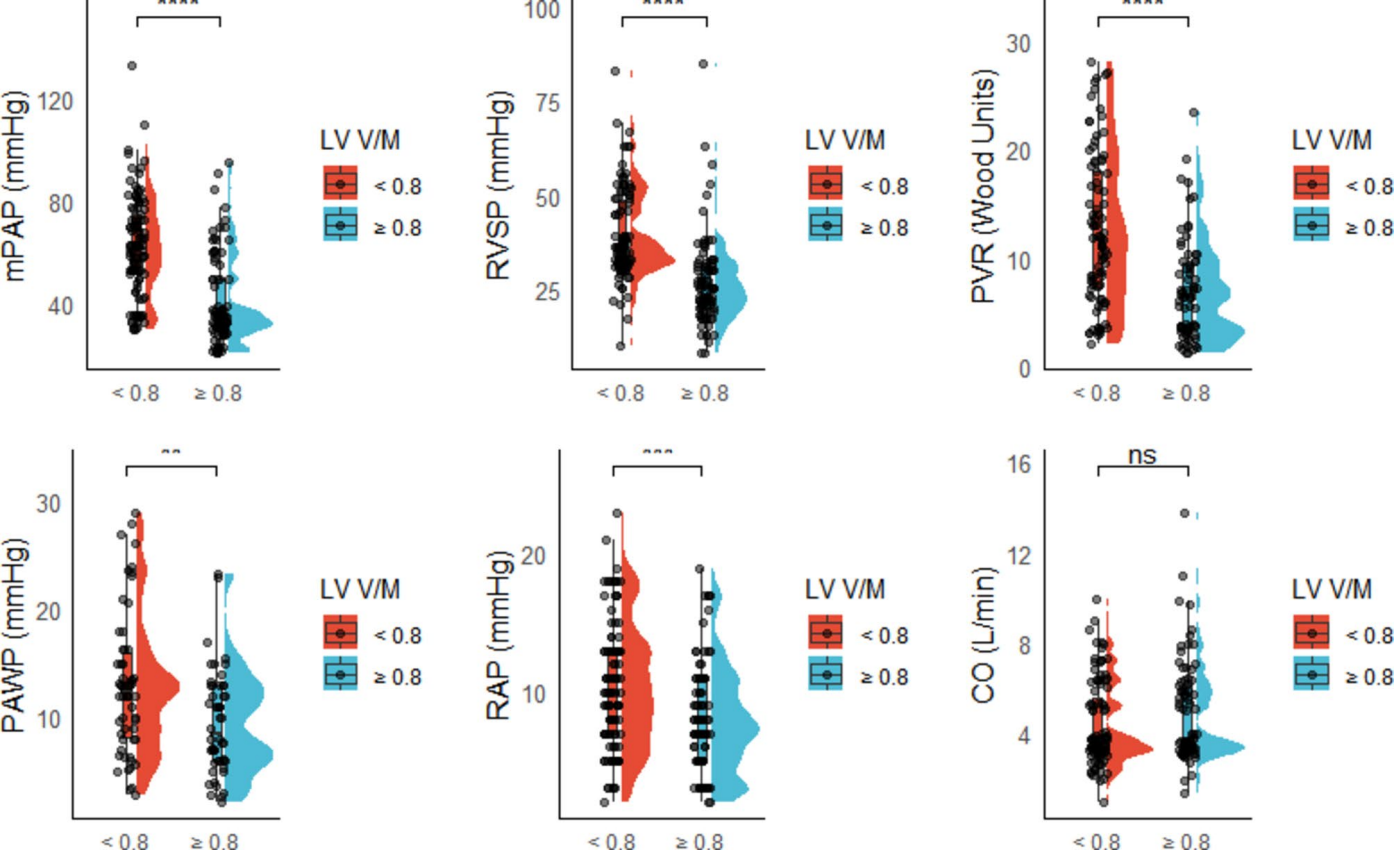
Median IQR plots comparing hemodynamic parameters with and without LV underfilling, highlighting central tendency and variability. Note: CO: cardiac output; mPAP: mean pulmonary artery pressure; PVR: pulmonary vascular resistance; PCWP: pulmonary capillary wedge pressure; RAP: right atrial pressure; RVSP: right ventricular systolic pressure.

**Fig. 5:**
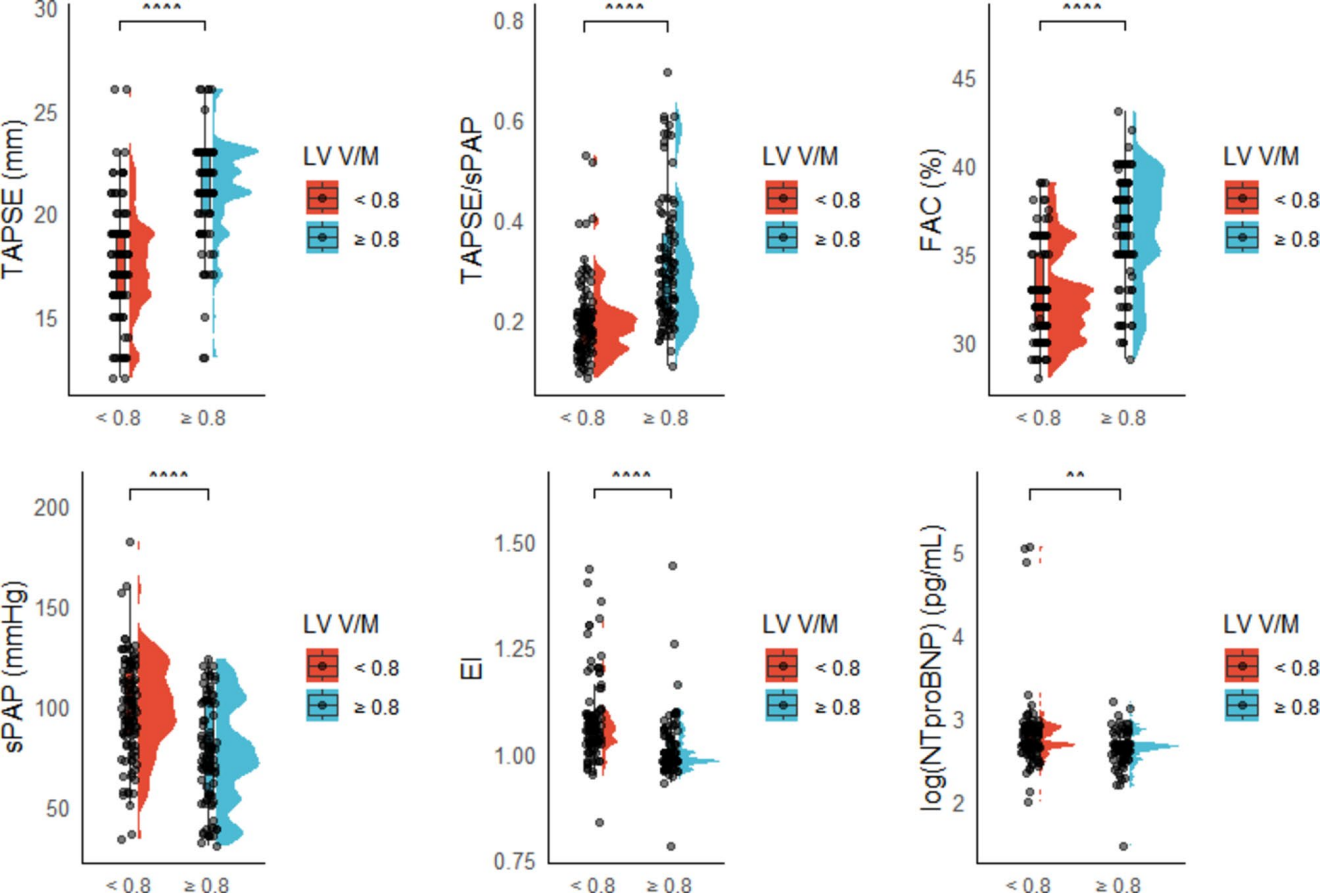
Median with IQR plots comparing RV-functional parameters with and without LV underfilling, highlighting the central tendency and variability. Note: FAC: fractional area change; sPAP: systolic pulmonary artery pressure; TAPSE: Tricuspid annular plane systolic excursion (TAPSE).

Key hemodynamic and RV functional parameters were compared between groups with and without LV underfilling. Patients with LV underfilling exhibited significantly higher mPAP, RVSP, and PVR (all p < 0.0001), as well as elevated RAP and PAWP (p = 0.04 and 0.01, respectively). However, CO showed no significant difference between the groups (Fig. 4). sPAP was significantly elevated in the LV underfilling group (p < 0.0001), reflecting hemodynamic strain from altered ventricular interaction. RV functional markers, including FAC, TAPSE, and the TAPSE/sPAP ratio, were significantly impaired in the LV underfilling group (all p < 0.0001), highlighting compromised RV performance in this condition^35^. RV-LV coupling, reflecting ventricular interdependence, was significantly higher in the LV underfilling group (p < 0.0001). This suggests RV dilation coupled with LV compression, indicating increased ventricular interdependence rather than functional synergy. Elevated right-sided pressures worsen LV filling, impairing true RV-LV coupling efficiency and highlighting maladaptive RV remodeling. The significantly higher LV-EI in the LV underfilling group (p < 0.0001) further supports the complex pathophysiological relationship between LV underfilling, RV dysfunction, and PAH severity (Fig. 5). These findings underscore the clinical importance of LV underfilling, affecting hemodynamic load, RV adaptation, and prognosis.

### Follow-up and survival analysis

**Fig. 6:**
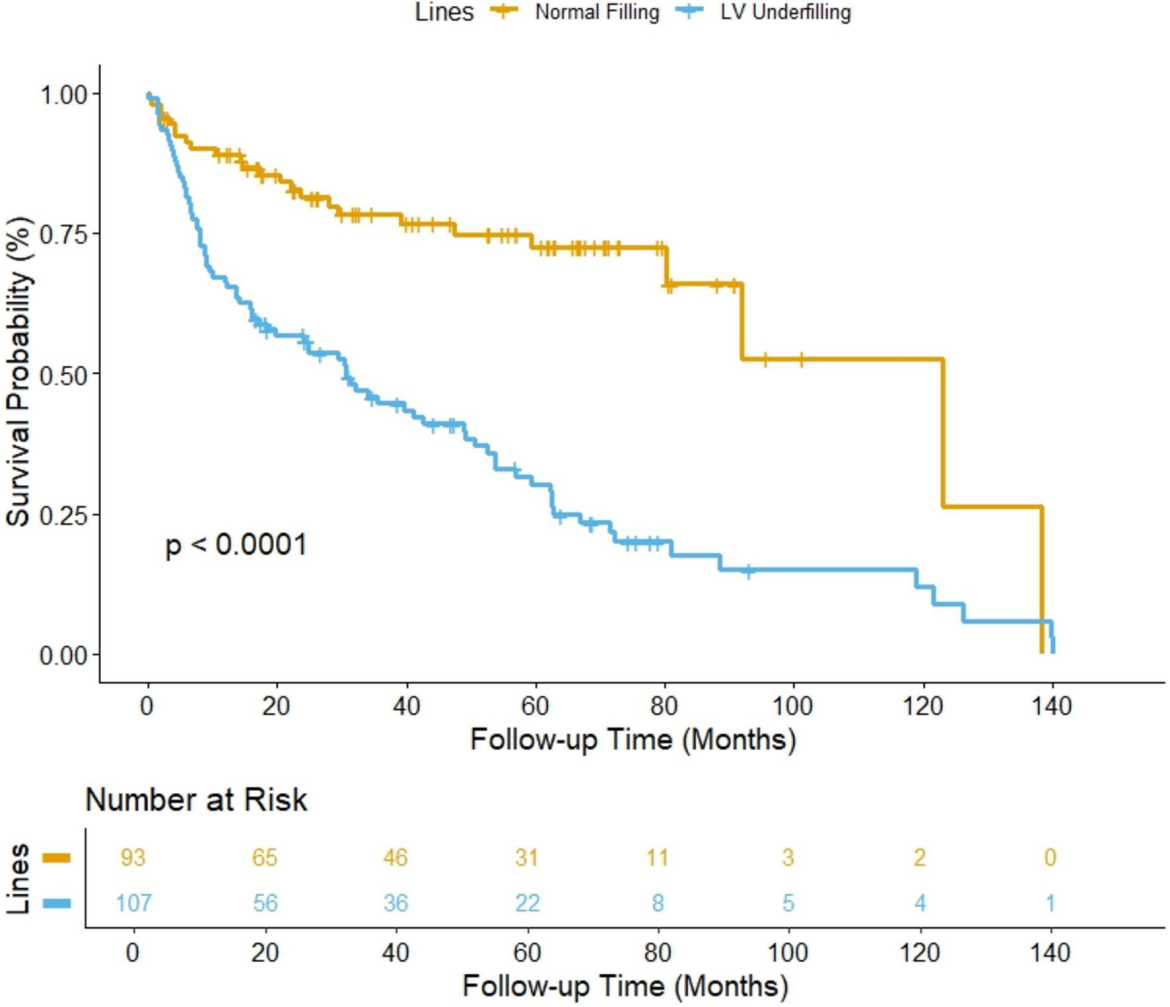
Kaplan–Meier survival curves comparing survival probability in patients with and without LV underfilling.

Over a median follow-up period of 29.8 months (IQR, 12.2–62.2 months), 114 patients experienced an event of interest. Of these 11 patients (4.9%) experienced all-cause mortality, while 98 (51.3%) required rehospitalization due to HF or escalation of HF-related medication. Patients with preserved LV V/M, indicative of LV normal filling, had a considerably better event-free survival in PAH than those with reduced LV V/M (Fig. 6).

### Risk factor analysis and prediction modeling

In univariate Cox regression, several LV parameters were significantly associated with adverse outcomes (Table 3). LV underfilling had the strongest association (HR: 3.344, 95% CI: 2.121–5.271, p < 0.001), followed by the RV/LV-basal ratio (HR: 3.598, 95% CI: 2.047—6.325, p < 0.001). LV V/M showed a protective effect (HR: 0.374, 95% CI: 0.197–0.709, p = 0.003). LVEDD^(Lt-Rt)^ and LVEDD^(AP)^ were inversely associated with the outcomes (HR: 0.969, 95% CI: 0.944–0.995, p = 0.019; 0.971, 95% CI: 0.947–0.995, p = 0.017, respectively). LVEDV (ml) was also linked to adverse outcomes (HR: 0.99, 95% CI: 0.982–0.998, p = 0.021). The EI showed a strong association with adverse outcomes (HR: 6.951, 95% CI: 1.244–38.833, p = 0.028). LVESD^(Lt-Rt)^ and LVESD^(AP)^ were significantly associated with the outcomes (HR: 0.953, 95% CI: 0.909–0.999, p = 0.042; HR: 0.953, 95% CI: 0.907–0.999, p = 0.048, respectively). Other parameters showed no significant effect.

**Table 3:** Univariate Cox-regression analysis of LV parameters for outcomes of interest.

Variable	Coefficient (β)	HR (exp(β))	95% CI for HR	SE(β)	z-value	p-value
LV-underfilling	1.207	3.344	2.121–5.271	0.23	5.247	< 0.001
RV/LV-basal (mm)	1.28	3.598	2.047–6.325	0.287	4.466	< 0.001
LV V/M (ml/g)	–0.982	0.374	0.197–0.709	0.328	–2.991	0.003
LVEDD^(AP)^ (mm)	–0.03	0.971	0.947–0.995	0.012	–2.378	0.017
LVEDD^(Lt-Rt)^ (mm)	–0.032	0.969	0.944–0.995	0.013	–2.342	0.019
LVEDV (ml)	–0.01	0.99	0.982–0.998	0.004	–2.308	0.021
EI	1.939	6.951	1.244–38.833	0.883	2.196	0.028
LVESD^(Lt-Rt)^ (mm)	–0.049	0.953	0.909–0.999	0.024	–2.034	0.042
LVESD^(A-P)^ (mm)	–0.048	0.953	0.907–0.999	0.024	–1.978	0.048
LVESV (mL)	–0.021	0.98	0.952–1.009	0.014	–1.43	0.153
PA/AO (mm)	0.23	1.258	0.553–2.860	0.419	0.548	0.583
LV-mass (g)	0.001	1.001	0.992–1.009	0.004	0.119	0.905
LA^(Lt-Rt)^ (mm)	–0.001	0.999	0.974–1.024	0.013	–0.06	0.952

CI: confidence interval; EI: eccentricity index; HR: hazard ratio; LA^(Lt-Rt)^: left atrium (left–right); LV: left ventricle; LVEDD^(AP)^: left ventricular end-diastolic diameter (antero-posterior); LVEDD^(Lt-Rt)^: left ventricular end-diastolic diameter (left–right); LVEDV: left ventricular end diastolic volume; LVESD^(AP)^: left ventricular end systolic diameter (antero-posterior); LVESD^(Lt-Rt)^: left ventricular end systolic diameter (left–right); LVESV: left ventricular end-systolic volume; LV V/M: LV volume-to-mass ratio; PA/AO: pulmonary artery to aorta ratio.

Multivariate Cox-regression analysis revealed that LV underfilling was strongly associated with adverse outcomes (HR: 5.323, 95% CI: 3.054–9.280, p < 0.001). Similarly, elevated RV/LV-basal ratio and LVEDD^(Lt-Rt)^ were significantly linked to a higher risk of adverse events (HR: 1.966, 95% CI: 1.066–3.626, p = 0.03; HR: 1.071, p = 0.0005). These findings highlight the RV/LV ratio and LV underfilling as key predictors of unfavorable clinical outcomes. Conversely, LVESD^(Lt-Rt)^ (HR: 0.954, 95% CI: 0.895–1.018, p = 0.154) and LVESD^(AP)^ (HR: 0.939, 95% CI: 0.872–1.011, p = 0.093) showed no significant associations with adverse events. (Table 4).

**Table 4:** Multivariate Cox-regression analysis of LV parameters for outcomes of interest.

Variable	Coefficient (β)	HR (exp(β))	95% CI for HR	SE(β)	z-value	p-value
LV underfilling	1.672	5.323	3.054–9.280	0.283	5.899	< 0.001
RV/LV-basal (mm)	0.676	1.966	1.066–3.626	0.312	2.165	0.03
LVEDD^(Lt-Rt)^ (mm)	0.069	1.071	1.030–1.114	0.02	3.441	0.0005
LVESD^(Lt-Rt)^ (mm)	–0.046	0.954	0.895–1.018	0.032	–1.424	0.154
LVESD^(AP)^ (mm)	–0.062	0.939	0.872–1.011	0.037	–1.675	0.093

LV: left ventricle; LVEDD^(Lt-Rt)^: left ventricular end-diastolic diameter (left–right); LVESD^(AP)^: left ventricular end-systolic diameter (anteroposterior); LVESD^(Lt-Rt)^: left ventricular end-systolic diameter (left–right). RV: right ventricle.

### Current LV model performance metrics

**Fig. 7:**
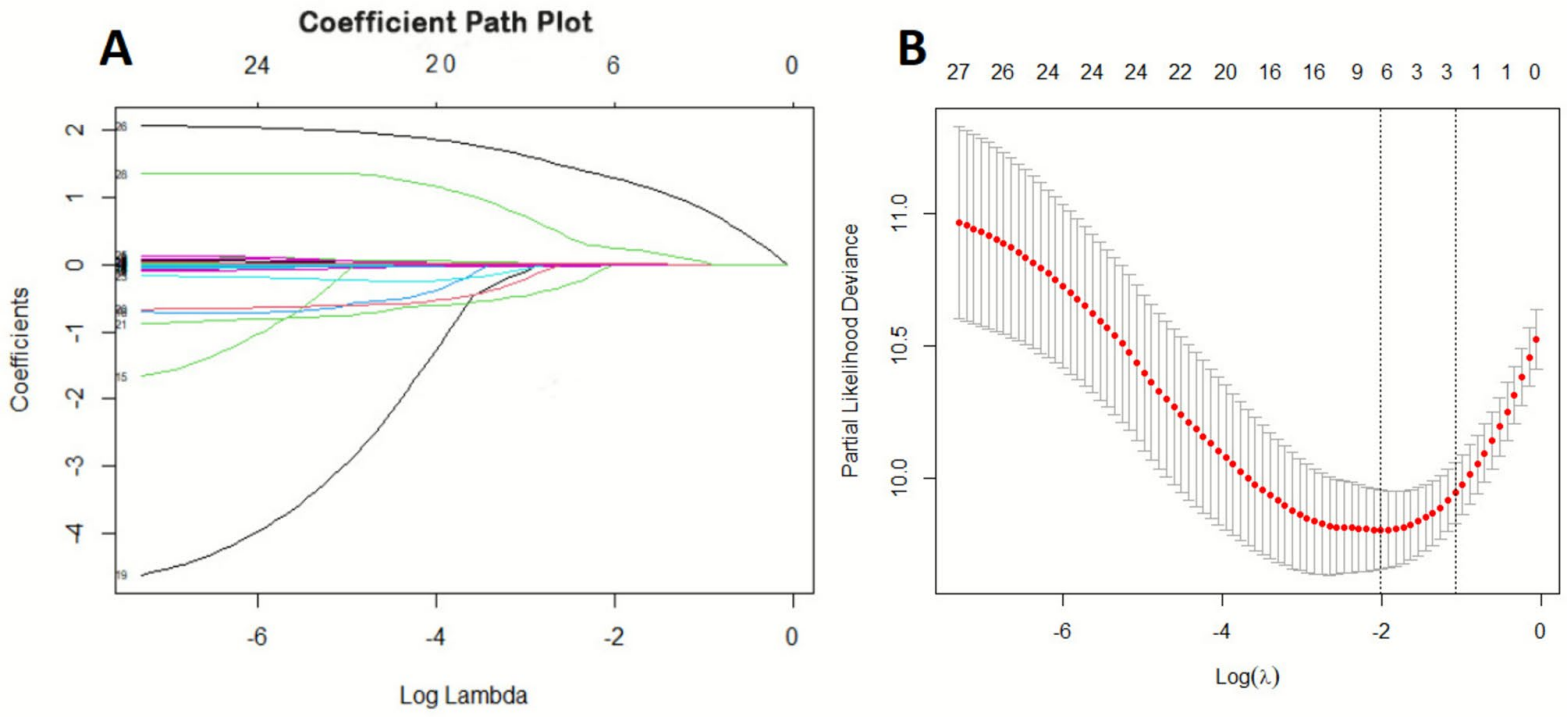
LASSO regression for predictive feature selection. (**A**) Coefficient paths as a function of the log of Lambda, illustrating the shrinkage of coefficients with increasing regularization strength; (**B**) Cross-validation error (mean squared error or deviance) as a function of the log of Lambda, with the optimal Lambda.

**Fig. 8:**
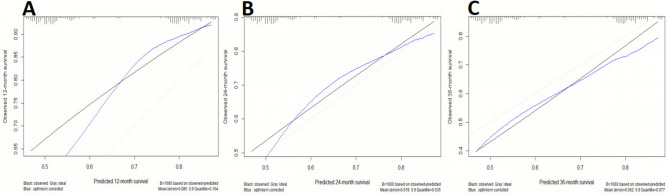
Calibration plot for the predicted versus observed survival. (**A**) 12 months, (**B**) 24 months, and (**C**) 36 months.

**Fig. 9:**
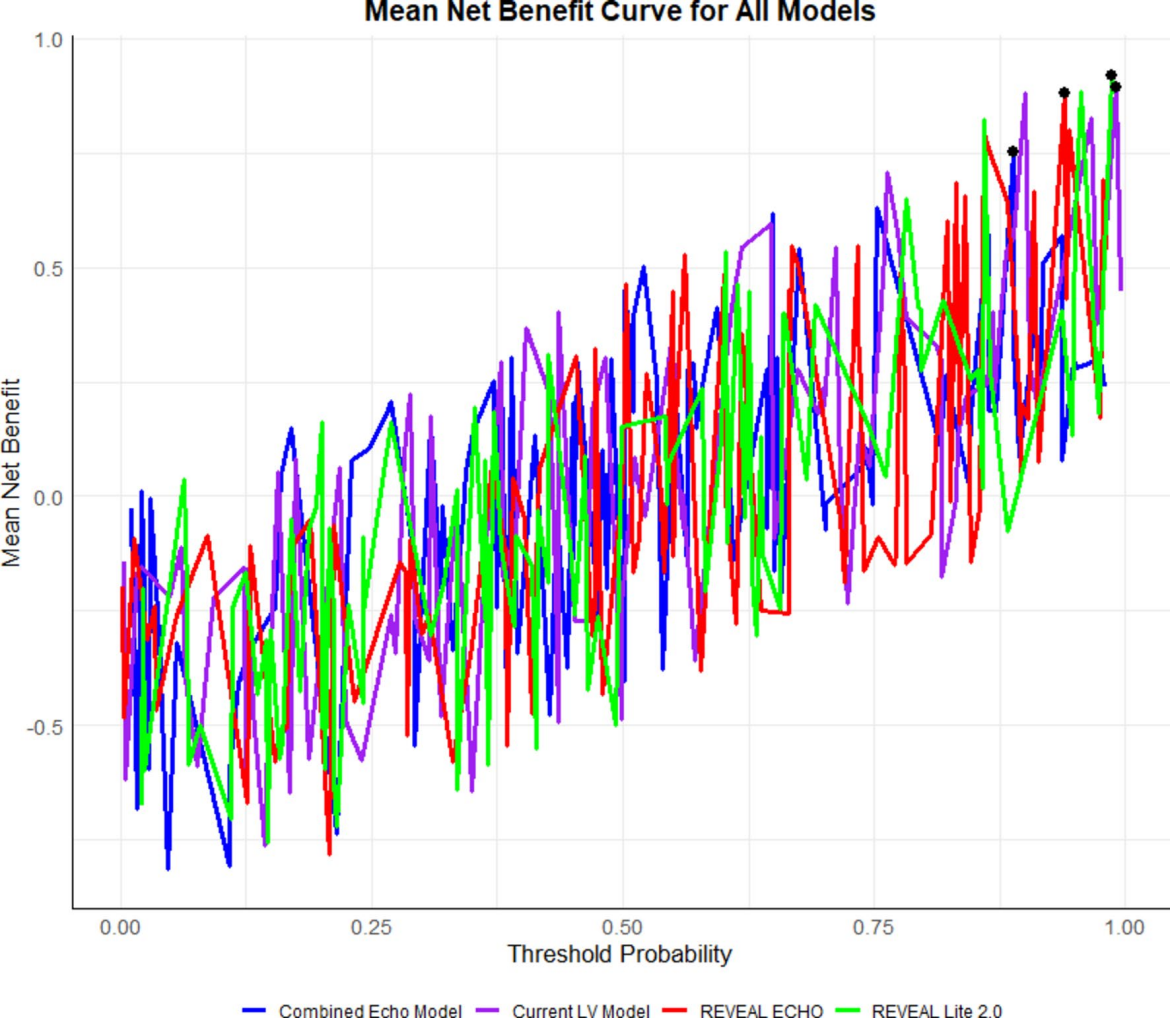
DCA evaluates the clinical utility of REVEAL-Lite 2.0, REVEAL-ECHO, the current LV model, and the combined echo model by quantifying net benefit across a range of threshold probabilities for decision-making. The X-axis represents the threshold probability at which a clinical intervention would be triggered, and the Y-axis represents the mean net benefit compared with “treat all” and “treat none” strategies.

LASSO regression identified predictive variables for adverse events in the LV Model (Fig. 7A–B), selecting five non-zero predictors for final model development. The model demonstrated strong discrimination (Mean C-index: 0.716 ± 0.06; AIC: 959.4; BIC: 975.8; Table 5). Calibration curves showed moderate accuracy at 12 months (mean error: 0.085; 0.9 quantile: 0.104; Fig. 8A), improved accuracy at 24 months (mean error: 0.018; 0.9 quantile: 0.035; Fig. 8B), but increased variability at 36 months (mean error: 0.062; 0.9 quantile: 0.77; Fig. 8C). Net benefit increased with higher threshold probabilities across all models, reflecting improved predictive accuracy under stricter criteria. REVEAL-Lite 2.0 performed consistently across thresholds, while REVEAL-ECHO and the current LV model showed greater net benefits at lower thresholds. The combined-Echo model also maintained comparable overall performance, supporting its clinical utility (Fig. 9).

**Table 5:** Model performance matrices.

Model	Mean C-index (± SD)	AIC	BIC
REVEAL-Lite 2.0	0.585 ± 0.07	1012.7	1015.5
REVEAL-ECHO	0.717 ± 0.11	985.1	987.8
Current LV-model	0.716 ± 0.06	959.4	975.8
Combine-Echo model	0.739 ± 0.05	960.3	979.4

AIC: Akaike Information Criterion; BIC: Bayesian Information Criterion.

### Current LV model vs established models

Four predictive models for adverse outcomes in PAH patients were developed: REVEAL-Lite 2.0 (The abridged version of REVEAL 2.0 calculator), REVEAL-ECHO, the current LV model, and the combined-Echo model (REVEAL-ECHO plus current LV model). REVEAL-Lite 2.0 employed the validated REVEAL risk score; REVEAL-ECHO included four echocardiographic measures, while the current LV model relied solely on LV parameters. The current LV model outperformed established models, exhibiting superior discrimination versus REVEAL-Lite 2.0 (Mean C-index: 0.716 ± 0.06 vs. 0.585 ± 0.07). Combined-Echo model, integrating RV and LV parameters, further improved predictive accuracy (Mean C-index: 0.739 ± 0.05). AIC and BIC values mirrored this trend: REVEAL-Lite 2.0 had the highest values (1012.7, 1015.5), indicating lower accuracy, while the REVEAL-ECHO (AIC: 985.1, BIC: 987.8) and the current LV model (AIC: 959.4, BIC: 975.8) performed better. The combined-Echo model slightly surpassed the current LV model in both criteria (AIC: 960.3, BIC: 979.4) (Table 5).

**Fig. 10:**
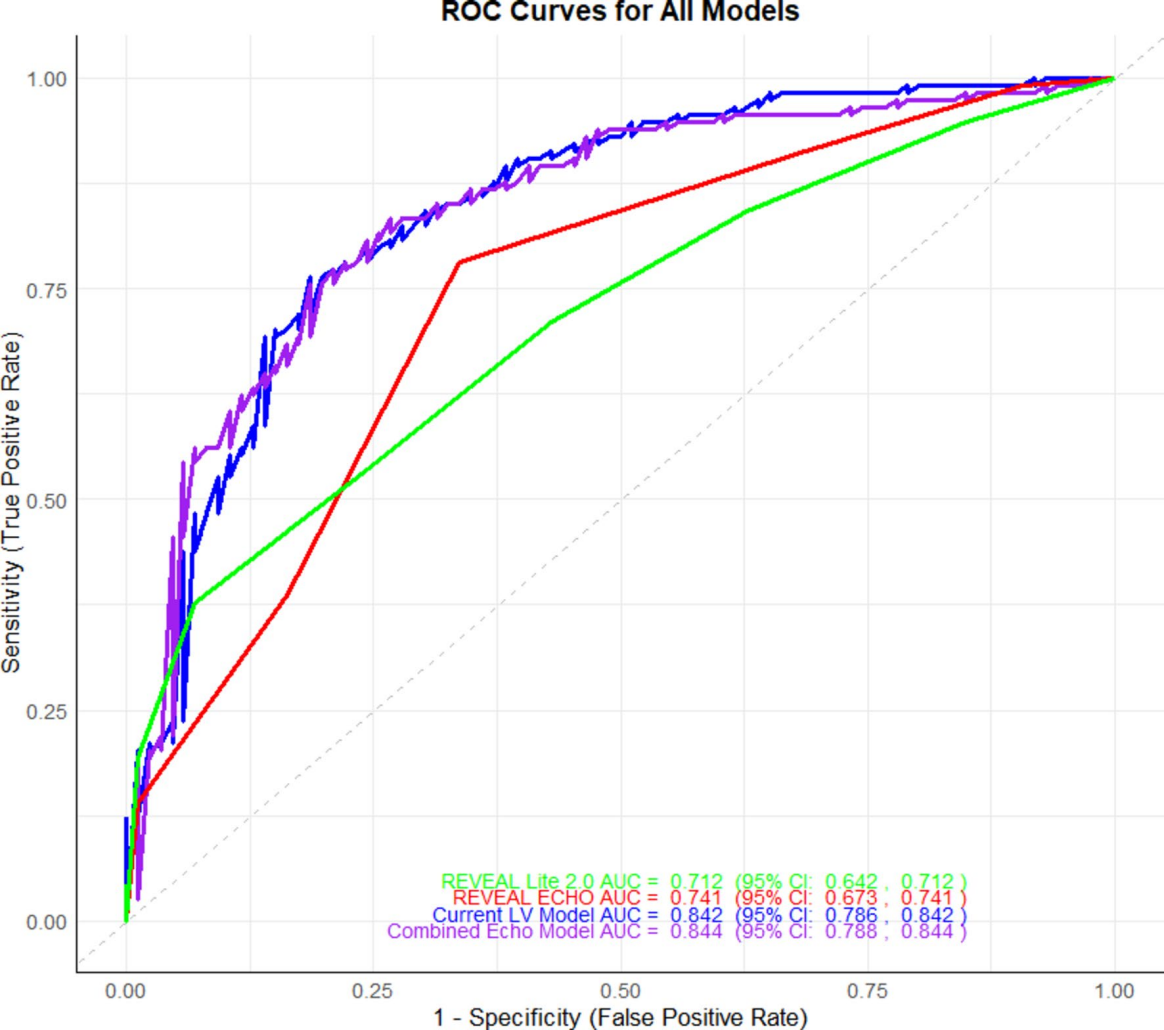
ROC curves illustrating the performance of REVEAL-Lite 2.0, REVEAL-ECHO, the current LV model, and the combined echocardiographic model. The x-axis represents the false positive rate (1 – specificity), and the y-axis represents the true positive rate (sensitivity). The area under the curve (AUC) is reported for each model, with corresponding 95% confidence intervals indicated in the legend.. AUC: area under the curve; CI: confidence interval.

NRI and IDI analyses demonstrated significant model performance improvements in reclassification and discrimination. The current LV model showed no reclassification gain over REVEAL-Lite 2.0 (NRI: 0) but significantly improved discrimination (IDI: 2.285). The combined-Echo model yielded significant gains in both NRI (0.059) and IDI (2.651), highlighting the advantage of integrating RV and LV data. Compared to REVEAL-ECHO, the combined-Echo model also moderately improved NRI (0.166) and IDI (2.344). These results indicate that combining RV and LV parameters significantly improves reclassification and discrimination for predicting adverse outcomes, outperforming individual models (Table 6). ROC analysis (Fig. 10) showed strong discrimination for the current LV model (AUC: 0.842, 95% CI: 0.786 − 0.842) and combined-Echo model (AUC: 0.844, 95% CI: 0.788–0.844). This underscores that integrating LV parameters with REVEAL-ECHO enhances predictive accuracy, highlighting the benefit of combining comprehensive echocardiographic data for risk stratification.

**Table 6:** Net Reclassification Improvement (NRI) and Integrated Discrimination Improvement (IDI) for Model Comparisons with 95% Confidence Intervals.

Model Comparison	NRI	95% CI		IDI	95% CI	
		**Lower**	**Upper**		**Lower**	**Upper**
REVEAL-ECHO vs. REVEAL-Lite 2.0	0.2231	0.3325	0.1027	0.4955	0.6824	0.1319
Current LV-model vs. REVEAL-Lite 2.0	0	0	0	2.2847	2.7971	1.7879
Combined-Echo model vs. REVEAL-Lite 2.0	0.0591	0.1111	0.0128	2.6514	3.2704	2.0185
Current LV-model vs. REVEAL-ECHO	0.225	0.3381	0.1144	1.9693	2.4624	1.4934
Combined-Echo model vs. REVEAL-ECHO	0.1661	0.2736	0.0636	2.3435	2.9171	1.7828
Combined-Echo model vs. Current LV-model	0.0581	0.1154	0.0118	0.3768	0.816	0.0098

IDI: Integrated Discrimination Improvement, NRI: Net Reclassification Improvement.

### Reproducibility

Reproducibility analysis showed good to excellent agreement for all LV parameters (Table 7). Intra-observer was excellent with low variability, as reflected with mean percentage differences ranging from 2.11% to 5.02% and ICC values 0.93 (95%CI: 0.87–0.97) to 0.98 (95%CI: 0.97–0.99). Inter-observer variability was slightly higher but remained within acceptable limits, with percentage differences ranging from 7.6% to 13.2% and ICC values between 0.81 (95% CI: 0.62–0.91) and 0.95 (95% CI: 0.92–0.98). These findings confirm the reliability of the LV measurements used in the current model.

**Table 7:** Intra- and inter-observer variability and intraclass correlation coefficients (ICC) for left ventricular parameters used in the current LV model.

Variable	Intra-Observer		Inter-observer	
	**% Variability (Mean ± SD)**	**ICC (95% CI)**	**% Variability (Mean ± SD)**	**ICC (95% CI)**
LV V/M (ml/g)	5.02 ± 5.77	0.93 (0.87–0.97)	9.05 ± 7.17	0.90 (0.69–0.96)
RV/LV-basal (mm)	4.47 ± 4.66	0.98 (0.97–0.99)	7.6 ± 9.43	0.95 (0.92–0.98)
LVEDD^(Lt-Rt)^ (mm)	2.11 ± 3.78	0.95 (0.91–0.98)	7.92 ± 7.96	0.85 (0.72–0.93)
LVESD^(Lt-Rt)^ (mm)	3.91 ± 6.74	0.93 (0.87–0.97)	11.2 ± 10.6	0.85 (0.72–0.93)
LVESD^(A-P)^ (mm)	2.6 ± 5.05	0.96 (0.94–0.99)	13.2 ± 12.6	0.81 (0.62–0.91)

ICC, intra-class correlation coefficient; LV: left ventricle; LVEDD(Lt-Rt): left ventricular end-diastolic diameter (left–right); LVESD(AP): left ventricular end-systolic diameter (anteroposterior); LVESD(Lt-Rt): left ventricular end-systolic diameter (left–right). RV: right ventricle.

## Discussion

### Key findings summary

This is the first study to identify LV underfilling, defined by an LV V/M threshold of 0.8 ml/g, as a hallmark of advanced PAH and a robust independent predictor of poor outcomes driven by RV overload and ventricular interdependence. Key findings include: (i) LV underfilling showed a strong prognostic association, surpassing the RV/LV-basal ratio; (ii) Current LV model, incorporating LV parameters, outperformed REVEAL-Lite 2.0 and REVEAL-ECHO with a higher mean C-index and improved reclassification metrics (NRI, IDI); (iii) Combined-Echo model significantly enhanced risk stratification, demonstrating the added value of biventricular assessment. These results underscore the synergistic prognostic benefit of comprehensive ventricular evaluation beyond single-chamber models.

### Strengths of the current LV model

Compared to more conventional variants such as REVEAL-Lite 2.0 and REVEAL-ECHO with in our cohort, the current LV model offers several benefits. Its enhanced discriminatory capacity, as evidenced by its greater C-index in comparison to REVEAL-Lite 2.0 calculated in the same dataset, is one of its main advantages. More sophisticated risk categorization is made possible by the model’s incorporation of LV parameters, such as LV underfilling and RV/LV-basal ratio, especially for high-risk patients. Furthermore, the NRI and IDI findings demonstrate that the current LV model outperforms REVEAL-Lite 2.0 in terms of reclassification and model discrimination, showing that adding LV data improves prediction accuracy. The strength of combining RV and LV parameters in a predictive model was further supported by the combined-Echo model, which specifically demonstrated the best AUC and net benefit. The proposed model integrates REVEAL-ECHO with LV data.

### Mechanistic insights

Despite the profound embryological distinctions between the left and right ventricles, both adhere to the fundamental "laws of the heart."^36^ Acute afterload increase initially induces heterometric adaptation via ventricular dilation per Starling’s law, followed by a homeometric phase characterized by enhanced contractility per Anrep’s law, maintaining output without dilation or raised filling pressures and promoting adaptive concentric hypertrophy. A later shift back to heterometric remodeling signals maladaptive dilation and HF progression^36–40^. Previous studies identify RV concentric hypertrophy as an adaptive response linked to improved prognosis^18,40–42^. However, emerging evidence challenges the view that RV concentric hypertrophy is universally adaptive^19^, highlighting the need to consider ventricular interdependence in evaluating its functional and clinical significance^20^. Ventricular interdependence, the direct mechanical interaction between the two ventricles via the interventricular septum^23,38^, shared myocardial fibers, and the pericardium, has been proposed as a major determinant of LV underfilling in PAH and may influence RV adaptation to chronic afterload^38^. Through leftward septal displacement and increased pericardial restraint, RV dilation increases RV wall stress and alters septal mechanics, which can impair RV contractile reserve and effective RV-PA coupling^43^. While our study did not directly measure ventricular-vascular coupling ratio (VVCR), strain, or pericardial constraint, our findings of reduced LV volume with preserved LV mass are consistent with the pathophysiological framework^44,45^.

This study, to our knowledge, is the first to detail LV morphological adaptation in PAH patients using TTE. We observed progressive LV cavity reduction without a corresponding decrease in LV mass, indicating that LV underfilling, rather than true atrophy, drives remodeling in PAH^46^. Contrasting evidence indicates that reductions in LV volume may precede decreases in LV mass, reflecting a temporal dissociation between volumetric remodeling and structural atrophy during disease progression^47^. Prolonged pathological exposure results in a 10–20% reduction in end-diastolic volume and a 5–15% decrease in myocardial mass^47^ likely due to direct and indirect interventricular interactions that remodel LV morphology and function^48^. In PAH, these interactions extend beyond structural changes, with LV underfilling marking advanced disease and strongly predicting poor clinical outcomes. Our findings demonstrate that LV underfilling, quantified as the LVEDV/LV mass ratio, was strongly associated with adverse clinical outcomes in PAH. Although mechanistic causality cannot be inferred from our observational design, it remains speculative in the absence of direct VVCR measurements. Future work incorporating VVCR, RV strain, and invasive hemodynamic measures will be essential to clarify whether LV underfilling is primarily a surrogate marker of maladaptive remodeling or represents a potentially modifiable therapeutic target.

In PAH, elevated PVR leads to chronic RV pressure overload and progressive RV remodeling. As RV dilation and hypertrophy advance, ventricular interdependence becomes a key pathophysiological mechanism affecting LV filling. The interventricular septum shifts leftward, particularly during diastole, thereby impairing LV relaxation and reducing its compliance. In addition, the pericardial constraint imposed by an enlarged RV limits the ability of the LV to expand, resulting in LV underfilling despite preserved systolic function. This diastolic restriction reduces LVEDV, limiting preload and thereby decreasing stroke volume and systemic CO. The resulting low CO state can lead to reduced end-organ perfusion, fatigue, exercise intolerance, and contribute significantly to symptom burden and poor functional status in PAH patients.

Our findings demonstrate that LV underfilling may represent a central mechanism linking RV dysfunction to systemic hypoperfusion and multi-organ involvement, making it a powerful prognostic indicator in PAH. Our findings support the use of echocardiographic LV volumetric indices, such as the LVEDV/LV mass ratio, as a non-invasive surrogate of this maladaptive physiology. Importantly, this underfilling is not simply a secondary phenomenon but has direct clinical consequences, such as systemic hypoperfusion, exercise intolerance, and end-organ dysfunction. As such, reversing or preventing LV underfilling could represent a potential therapeutic target in PAH. For instance, therapies that reduce RV afterload (e.g., pulmonary vasodilators) may improve RV remodeling, reduce septal displacement, and thereby restore LV filling. Optimizing volume status and targeting RV function may also help mitigate interventricular constraint and enhance LV preload. Identifying patients with marked LV underfilling may also help stratify risk and guide intensified therapy or closer follow-up.

### LV underfilling and existing biomarkers

Although established traditional biomarkers of RV dysfunction, such as TAPSE^8,10,49^, RV free wall strain (RVFWS)^50^, RV-PA coupling^51–53^, RA size^54–57^, and NT-proBNP^6,58^ have been extensively studied and validated in PAH, LV underfilling provides exclusive prognostic information that complements these markers. We evaluated LV underfilling alongside established biomarkers to demonstrate its incremental prognostic value (Supplementary Table 1). As shown in Supplementary Table 2, LV underfilling improves risk stratification by integrating biventricular dynamics beyond conventional RV metrics. Unlike TAPSE, which overlooks RV-induced LV geometric changes, and RVFWS, which requires advanced imaging, LV underfilling is easily measured by standard echocardiography and captures ventricular interdependence and the mechanical effects of RV overload on LV filling. Incorporating LV underfilling into current prognostic models may enhance predictive accuracy and enable more personalized management in PAH.

### Clinical implication

**Fig. 11:**
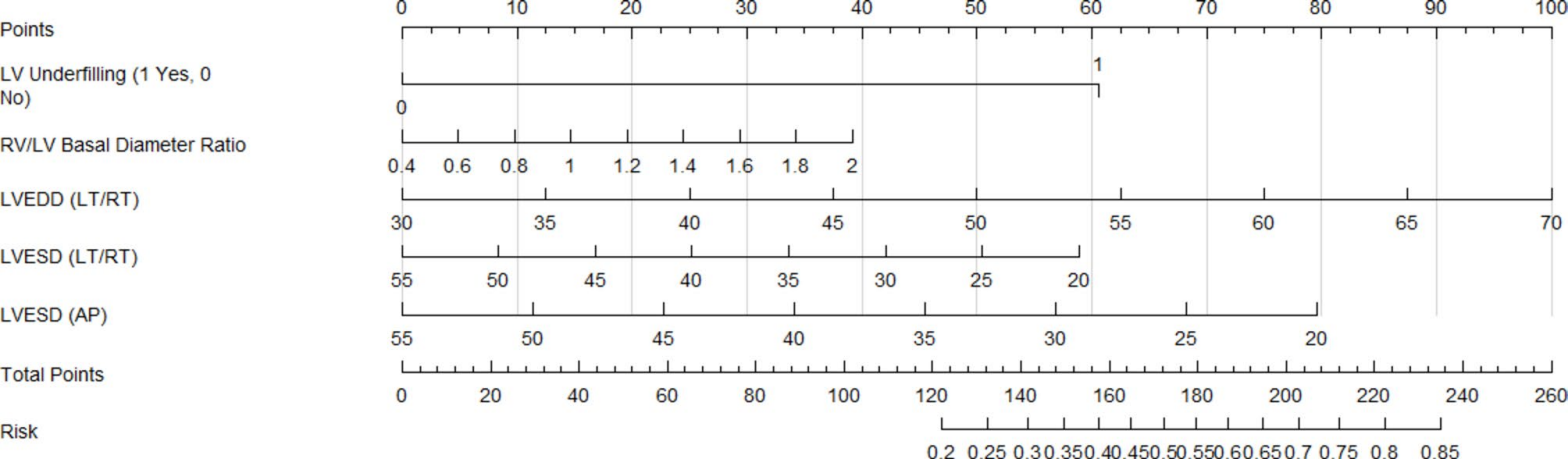
The nomogram visually represents the model’s predictions, where each predictor is assigned a score, and the total score is mapped to the probability of the outcome.

LV underfilling and the RV/LV-basal diameter offer simple, non-invasive metrics easily obtained via routine echocardiography. Incorporating these parameters into the current LV model alongside the REVEAL-ECHO model, beyond traditional RV-focused assessments, significantly enhances risk stratification accuracy in severe PAH. This integrated approach outperforms REVEAL-Lite 2.0 in patient discrimination and reclassification at 12, 24, and 36 months, enabling more precise identification of high-risk patients. By capturing LV involvement, it uncovers individuals potentially overlooked by RV-focused models, allowing clinicians to tailor interventions and initiate aggressive therapies earlier in those at greatest risk of adverse outcomes and disease progression. This clinically practical and cost-effective model leverages routine echocardiographic LV parameters for risk stratification. Combined with REVEAL-ECHO, the current LV model (Fig. 11) enhances hemodynamic and functional assessment, enabling personalized treatment strategies in severe PAH to improve long-term outcomes and patient management.

### Limitations

While our findings underscore the prognostic significance of LV underfilling, several limitations merit consideration. The RV/LV-basal diameter, used as a surrogate for RV-LV coupling, though conceptually sound, lacks prior validation and may not fully reflect the complex hemodynamics of ventricular interaction. The reliance on echocardiography, despite its accessibility, may be less precise than advanced modalities like CMR or invasive pressure–volume analysis. Moreover, the cross-sectional design precludes causal inference and temporal assessment. Future research should validate these results in larger, multicenter cohorts and investigate LV underfilling as a therapeutic target in PAH. Longitudinal studies employing serial echocardiography and advanced imaging are needed to elucidate ventricular remodeling dynamics and their clinical implications. While concentric LV remodeling has often been considered adaptive, our data caution that a reduced LV V/M may also reflect pathophysiologic underfilling, which is associated with adverse outcomes. We acknowledge that our study did not directly measure ventricular-vascular coupling (Ees/Ea) or detailed PV-loop indices of RV contractility and coupling, which are optimal for characterizing adaptive versus maladaptive RV remodeling. Derivation of the formal VVCR requires simultaneous high-fidelity pressure and volume measurements or validated single-beat methods that were not available for all patients in this retrospective cohort. Consequently, we interpreted LV V/M as a pragmatic, echocardiographic surrogate of LV underfilling and ventricular interdependence rather than a definitive mechanistic readout. Future prospective studies that combine echocardiographic and CMR with invasive pressure–volume assessment and advanced RV biomechanical indices (strain, wall stress, contractile reserve) are needed to determine whether impaired VVCR and interventricular decoupling mediate the transition from adaptive to maladaptive RV remodeling in PAH.

An important aspect of this study is that it evaluated LV structural and functional adaptation across a heterogeneous PAH population, including idiopathic pulmonary arterial hypertension, connective tissue disease-associated PAH, and congenital heart disease-associated PAH. While these subgroups differ in etiology, pathophysiology, and clinical course, they share the common hemodynamic consequence of chronic RV pressure overload and consequent LV underfilling due to ventricular interdependence. By analyzing these phenotypes together, we aimed to capture the overarching mechanistic relationship between RV pressure loading, LV preload limitation, and prognosis, thereby emphasizing LV underfilling as a potentially universal prognostic marker across PAH subtypes. Future studies with larger, etiology-specific cohorts will be important to confirm whether the strength of this association varies by PAH subtype. Finally, our comparison of the proposed model with REVEAL-Lite 2.0 and REVEAL-ECHO was performed using the same patient cohort that was used for model development. While this approach enables a direct head-to-head comparison within a consistent dataset, it may overestimate the comparative performance of the model due to potential optimism bias. Future work should validate this comparison in an independent external cohort to ensure generalizability.

## Conclusion

LV markers, including LV underfilling and the RV/LV-basal ratio, emerge as novel prognostic indicators in PAH. The newly developed current LV model, integrating these parameters, outperforms established tools like REVEAL-Lite 2.0 and REVEAL-ECHO in discrimination and risk reclassification. Combining the current LV model with REVEAL-ECHO further refines risk stratification, enabling more precise therapeutic decisions. Incorporating LV markers into prognostic frameworks offers a promising approach for personalized PAH management and improved patient outcomes.

In summary, the current LV model advances PAH prognosis by providing a robust, clinically applicable tool for risk assessment. Future studies should validate its utility in larger cohorts to establish long-term clinical impact.

## Supplementary Information

Supplementary Information 1.

Supplementary Information 2.

## Data Availability

The data that support the findings of this study are available from the authors, but restrictions apply to the availability of these data, which were used under license for the current study, and so are not publicly available. Data are, however, available from the authors upon reasonable request and with permission of the corresponding author.
