# Supplementary material for: Transthoracic echocardiography of left ventricular underfilling improves risk stratification in pulmonary arterial hypertension
Source: Sci Rep. 2025 Dec 4;15:45718. doi: 10.1038/s41598-025-28206-z (PMC12753818; doi:10.1038/s41598-025-28206-z)
Supplement: Supplementary file 1 — Supplementary Information 1. [file 41598_2025_28206_MOESM1_ESM.docx]

| **Supplementary Table 1. Incremental prognostic value of LV underfilling on traditional biomarkers in PAH.** | | | | | |  |
| --- | --- | --- | --- | --- | --- | --- |
| **Biomarker** | **Definition/Measurement** | **Prognostic value of PAH** | **Strengths** | **Limitations** | **Incremental Value of LV underfilling** | |
| TAPSE | Tricuspid annular plane systolic excursion (mm) measured by echocardiography | Predicting mortality and RV dysfunction | Non-invasive, widely available, and easy to measure | Reliable in severe tricuspid regurgitation and does not capture LV dynamics | Captures ventricular interdependence, which cannot be captured by TAPSE alone | |
| RVFWS | RV free wall longitudinal strain (%) measured by speckle-tracking echocardiography | Strong predictors of RV failure and adverse outcomes | Sensitive to early RV dysfunction, and provides detailed myocardial deformation | It requires advanced imaging and is less accessible in routine clinical practice | Reflects biventricular interaction, not just RV function | |
| RA Size | Right atrial size measured by echocardiography | Predicting mortality and RV dysfunction | Easy to measure, reflecting chronic RV pressure overload | Less specific to RV function influenced by atrial fibrillation or other factors | Provides direct insight into LV-RV coupling and septal shift | |
| NTproBNP | N-terminal pro-B-type natriuretic peptide (pg/mL) measured by blood test | Predicting mortality and disease progression | Widely available, strong prognostic value | Non-specific, influenced by other conditions (e.g., renal dysfunction) | Provide structural and functional insights to complement biochemical markers | |
| RV-to-LV ratio | The ratio of RV to LV basal diameter measured by echocardiography | Reflecting RV dilatation and LV compression (Current Study) | Simple and reflects ventricular interdependence | The structural measure does not assess function | Integrates the structural and functional aspects of ventricular interdependence | |
| 6MWD | Distance walked in 6 minutes (meters) | Correlates with survival and functional capacity | Simple functional measure of exercise capacity | Influenced by non-cardiac factors (e.g., musculoskeletal issues) | Provide objective imaging-based prognostic information | |
| LV Underfilling | LV volume-to-mass (V/M) ratio <0.8 mL/g, as measured by echocardiography | Predicts adverse outcomes (HR: 0.208, 95% CI: 0.094–0.505, p = 0.0007) (Current Study) | Reflects ventricular interdependence and RV-LV coupling | Requires precise echocardiographic measurement and is less reliable in terms of poor image quality | Prognostic value of integrating LV and RV dynamics to capture the impact of RV overload on LV geometry | |
| 6MWD: 6-min walk distance; HR: hazard ratio; LV: left ventricle; RA: right atrium; RVFWS: right ventricular free wall longitudnial strain; TAPSE; tricuspid annular plane systolic excurrsion; ln NT-proBNP; log NT-proBNP. | | | | | |  |
