# Supplementary material for: Transthoracic echocardiography of left ventricular underfilling improves risk stratification in pulmonary arterial hypertension
Source: Sci Rep. 2025 Dec 4;15:45718. doi: 10.1038/s41598-025-28206-z (PMC12753818; doi:10.1038/s41598-025-28206-z)
Supplement: Supplementary file 2 — Supplementary Information 2. [file 41598_2025_28206_MOESM2_ESM.docx]

| **Supplementary Table 2: Interaction analysis of LV underfilling with established prognostic markers in PAH.** | | | | |
| --- | --- | --- | --- | --- |
| **Prognostic Marker** | **Interaction Term** | **p-value for interaction** | **HR for LV underfilling (subgroup 1)** | **HR for LV underfilling (subgroup 2)** |
| WHO-FC | LV underfilling × WHO-FC | 0.01 | 0.64 (WHOFC-III) | 3.94 (WHOFC-IV) |
| TAPSE, mm | LV underfilling × TAPSE | 0.002 | 0.03 (TAPSE ≥ 17 | 0.25 (TAPSE < 17) |
| PVR, Wood | LV underfilling × PVR | 0.045 | 17.5 (PVR-4-8, wood) | 2.69 (PVR >8, wood) |
| Ci L/min/m2 | LV underfilling × CI | 0.427 | 6.69 (CI >2.5, L/min/m2) | 3.42 (CI <2.5, L/min/m2) |
| log(NTproBNP, pg/ml) | LV underfilling × log (NTproBNP ) | 0.021 | 1.65 (NTproBNP ≤1100 pg/ml) | 2.84 (NTproBNP >1100 pg/ml) |
| CI: cardiac Index; LV: left ventricle; PVR: pulmonary vascular resistance; TAPSE: tricuspid annular plane systolic excursion; WHOFC: World Health Organization functional class. | | | | |
